# Stroke in a resource-constrained hospital in Madagascar

**DOI:** 10.1186/s13104-017-2627-4

**Published:** 2017-07-24

**Authors:** Pål Sigurd Stenumgård, Miadana Joshua Rakotondranaivo, Olav Sletvold, Turid Follestad, Hanne Ellekjær

**Affiliations:** 1 Department of Geriatrics, St. Olavs Hospital, Postbox 3250, Sluppen, 7006 Trondheim, Norway; 2 Department of Neuromedicine and Movement Science, Faculty of Medicine and Health Sciences, Norwegian University of Science and Technology (NTNU), Postbox 8905, 7491 Trondheim, Norway; 3 Faculty of Medicine, University of Antananarivo, B.P 375, 101 Antananarivo, Madagascar; 4 Department of Public Health and Nursing, Faculty of Medicine and Health Sciences, Norwegian University of Science and Technology (NTNU), Postbox 8905, 7491 Trondheim, Norway; 5 Stroke Unit, St. Olavs Hospital, Postbox 3250, Sluppen, 7006 Trondheim, Norway

**Keywords:** Stroke, Stroke assessment scales, Stroke management protocol, Stroke outcomes, Mortality, Low-income country, Madagascar, Sub-Saharan Africa

## Abstract

**Background:**

Stroke is reported as the most frequent cause of in-hospital death in Madagascar. However, no descriptive data on hospitalized stroke patients in the country have been published. In the present study, we sought to investigate the feasibility of collecting data on stroke patients in a resource-constrained hospital in Madagascar. We also aimed to characterize patients hospitalized with stroke.

**Methods:**

We registered socio-demographics, clinical characteristics, and early outcomes of patients admitted for stroke between 23 September 2014 and 3 December 2014. We used several validated scales for the evaluation. Stroke severity was measured by the National Institutes of Health Stroke Scale (NIHSS), disability by the modified Rankin Scale (mRS), and function by the Barthel Index (BI).

**Results:**

We studied 30 patients. Sixteen were males. The median age was 62.5 years (IQR 58–67). The NIHSS and mRS were completed for all of the patients, and BI was used for the survivors. Three patients received a computed tomography (CT) brain scan. The access to laboratory investigations was limited. Electrocardiographs (ECGs) were not performed. The median NIHSS score was 16.5 (IQR 10–35). The in-hospital stroke mortality was 30%. At discharge, the median mRS score was 5 (IQR 4–6), and the median BI score was 45 (IQR 0–72.5).

**Conclusions:**

Although the access to brain imaging and supporting investigations was deficient, this small-scale study suggests that it is feasible to collect essential data on stroke patients in a resource-constrained hospital in Madagascar. Such data should be useful for improving stroke services and planning further research. The hospitalized stroke patients had severe symptoms. The in-hospital stroke mortality was high. At discharge, the disability category was high, and functional status low.

## Background

Cerebrovascular disease is the second leading cause of years of life lost worldwide [1]. The burden of stroke varies considerably between countries, but low-income countries are the most affected [2, 3].

Madagascar is a low-income country in sub-Saharan Africa (SSA), with a population of 24.2 million [4]. With more than 92% of its population living on less than US$2 a day, Madagascar is one of the poorest countries in the world [5]. The prevalence of human immunodeficiency virus (HIV) is estimated to be 0.4% [6]. The Global Burden of Disease 2013 Study suggests that the mortality rates of ischemic and hemorrhagic strokes in Madagascar are among the highest in the world [7]. The Ministry of Health in Madagascar reports that stroke is the most frequent cause of in-hospital death in the country [8]. Based on a search in PubMed, there are no published data on hospitalized stroke patients in Madagascar.

Community-based data on stroke patients are scarce in the developing region of SSA. However, hospital-based data on stroke are available from low-income countries, such as Ethiopia, the Gambia, Malawi, Mozambique, and Uganda [9–13]. These five studies were mostly carried out in referral hospitals, but none had a stroke unit [14]. The age (median or mean) of these patients was in the sixth or seventh decennium [9–13]. The majority of the patients had severe stroke symptoms measured by the National Institutes of Health Stroke Scale (NIHSS) or the Scandinavian Stroke Scale [9–11, 13]. Hypertension as a risk factor ranged from 48% in the Gambia to 86.2% in Mozambique [10, 12]. Between 25.8 and 43.6% had hemorrhagic stroke in the studies, in which the pathological stroke type was confirmed by brain imaging or autopsy [9, 11–13]. In-hospital stroke mortality ranged from 14.7% in Ethiopia to 41% in the Gambia [9, 10]. The in-hospital stroke mortality rates from these countries were considerably higher than that reported from high-income countries [15].

Developments of modern stroke services have been done in high-income countries [16]. This raises the question whether such care is relevant and applicable to resource-constrained settings. This issue becomes even more urgent as new imaging techniques and endovascular procedures are introduced to stroke services in high-income countries [17, 18]. It is therefore crucial to explore the possibilities of carrying out studies on stroke care in the poorest countries in the world.

Our study aimed to investigate the feasibility of collecting data on stroke patients in a resource-constrained hospital in Madagascar. We also wanted to map the local settings in case of planning a larger study on stroke care. We focused primarily on sampling data using validated scales for stroke evaluation. Furthermore, we wanted to describe the characteristics and early outcomes of patients hospitalized with stroke. Finally, we made an estimate of the prevalence of stroke in the Department of Medicine.

## Methods

### Study setting

The study took place at the Andranomadio Lutheran Hospital, one of four hospitals in Antsirabe, the main city of the Vakinankaratra region with a population of 1.9 million [19]. The Department of Medicine at the Andranomadio Lutheran Hospital had 53 beds for patients aged 15 years or older. The department was staffed with four medical doctors and 15 nurses. The staff was also in charge of the emergency room and an outpatient clinic. A physiotherapist could be called from outside the hospital. Family members were responsible for feeding the patient, turning the patient in bed, and assisting with urinary and bowel elimination. Blood pressure was measured manually. The hospital had a simply equipped laboratory for clinical chemistry. However, the blood hemoglobin analyzer was not working for eight out of the 10 weeks during the study period. HIV tests were not performed because they lacked reagents. Blood lipids were not routinely measured. The only electrocardiography (ECG) device was out of order. An X-ray machine and equipment for basic ultrasound examinations were available. A computed tomography (CT) scan could be performed by transferring to another hospital (Centre Hospitalier de Référence Régional de Vakinankaratra). No laboratory in Antsirabe performed the test for the International Normalized Ratio (INR) for prothrombin time.

All of the hospitals in Antsirabe demanded patient payment. In the Andranomadio Lutheran Hospital, the fee for a stay was approximately US$1 a day. Additionally, the patients had to pay for clinical chemical assays, diagnostic imaging, medical disposables, and drugs. The price for a CT brain scan was US$80. There was no universal health coverage.

Most patients presented directly to the hospital without any referral document. Access to ambulances was very limited. Therefore, the patients were brought to the hospital in pulled rickshaws, private cars, or public mini-buses. When there was a need to transfer a patient to another hospital, a pulled rickshaw was the most common mode of transport.

### Patient selection

A stroke was defined according to the World Health Organization (WHO) clinical criteria as “rapidly developing signs of focal or global disturbance of cerebral function, leading to death or lasting longer than 24 h with no apparent cause other than vascular” [20].

Patients admitted for stroke (first or recurrent) between 23 September 2014 and 3 December 2014 were considered eligible for the study. We included patients with first time hospitalization due to current stroke regardless of when the symptoms had started. We excluded patients with symptoms suggestive of a subarachnoid hemorrhage or central nervous system infections, patients with paresis after a seizure, and patients who were readmitted with post-stroke complications. All of the patients were seen by PSS and MJR or another native Malagasy-speaking interpreter at recruitment.

### Data collection

PSS and MJR collected the data in cooperation with the staff. Socio-demographics, risk factors, and disability prior to current stroke were based on an interview with the patient or a next of kin and a review of the available medical records. PSS and MJR conducted the clinical scales and stroke classifications by bedside evaluations with the patients. Swallowing function was evaluated by testing or self-reporting. Blood pressure readings and laboratory results were obtained from medical records and CT scan data from radiology reports. The number of patients hospitalized, length of hospital stay, and in-hospital mortality were collected from the ward registries.

### Clinical scales

We measured stroke severity by the NIHSS with a score ranging from 0 to 40 (a high score corresponds to more severe stroke symptoms) [21]. We calculated the Siriraj Stroke Score (SSS) to indicate the pathologic stroke type (a score above 1 indicates hemorrhagic stroke, a score below −1 indicates ischemic stroke, and a score between 1 and −1 represents an equivocal result) [22]. Regardless of the stroke subtype, the Oxfordshire Community Stroke Project (OCSP) classification was used for the lesion location and its extension [23]. Disability was measured by the modified Rankin Scale (mRS) with a score ranging from 0 to 6 (a high score suggests more disability and an mRS of 6 indicates death) [24]. The level of independent function in activities of daily living (ADL) and mobility were measured by the Barthel Index (BI) with a score ranging from 0 to 100 (a high score suggests increased independence) [25]. We applied English versions of the scales.

### Definitions of clinical characteristics and risk factors

The level of consciousness was categorized as “awake” if the patient was alert and oriented, “drowsy” if the patient had reduced wakefulness, and “no response” if the patient was unarousable. Swallowing function was classified as “impaired” if there were any problems with swallowing a spoonful of water or cooked rice, and as “not testable” if the level of consciousness was considerably depressed. Mobilization was defined as getting the patient out of bed, to sit, stand, or walk [26]. We recorded a few neurological complications (stroke progression and seizures) and some complications of immobility (chest infection, urinary tract infection, and bedsore) [27]. Stroke progression was defined as an increase in NIHSS score of ≥2 points or death occurring within 72 h of onset with no other explanation than the stroke itself [28]. In-hospital stroke mortality was defined as the proportion of stroke patients who died (irrespective of cause) during the stay.

Hypertension was defined as known blood pressure >140/90 mmHg before admission or current use of antihypertensive drugs. Blood pressure readings were categorized as low (<100/60 mmHg), normal (100/60–140/90 mmHg), or high (>140/90 mmHg). Diabetes was defined as a previously documented diagnosis, a fasting plasma glucose ≥7.0 mmol/L, or a random plasma glucose ≥11.1 mmol/L [29]. The diagnosis was never based on a single glucose determination. Plasma glucose level was categorized as low (<4.0 mmol/L), normal (4.0–6.9 mmol/L), intermediate (7.0–11.0 mmol/L), or high (≥11.1 mmol/L). Tobacco smoking was defined as “currently” if the patient smoked occasionally or daily in the last month and as “previously” if the patient quit smoking more than 1 month before the current stroke. Information on previous stroke and intermittent claudication was self-reported or specified in medical records.

### Statistical analysis

The data were summarized using the median and interquartile range (IQR) for continuous data and frequencies and percentages for categorical data. The statistical analysis was performed using IBM SPSS Statistics version 22.

An estimate of the period prevalence of stroke (first or recurrent) in the Department of Medicine for October and November 2014 was calculated as the number of patients with stroke discharged divided by the total number of patients discharged (alive or dead) during these 2 months.

## Results

Thirty-one patients were admitted with a diagnosis of stroke. One patient died before consent was obtained. We studied the remaining 30 patients, composed of 16 males and 14 females, with a median age of 62.5 years (IQR 58–67). All of the patients were living at home at the time of admission, and the median mRS score prior to the current stroke was 1 (IQR 0–1). The time between the onset of symptoms and admission varied greatly. Nineteen patients were admitted within 24 h, six patients between 1 and 7 days, and five patients later than 7 days after onset. The median prehospital time lapse was 12 h (range 0–38 days), and the median time from admission to study inclusion was 5 h (range 1–40). At inclusion, the NIHSS was performed in all of the patients. Five patients were unclassifiable according to the OCSP classification. During the stay, the SSS was completed for all of the patients. At discharge, an mRS score was determined for all of the patients, and a BI score was determined for the survivors.

Table 1 summarizes the baseline socio-demographic and clinical characteristics of the study participants. The median NIHSS score was 16.5 (IQR 10–35). Twenty-two patients presented with a NIHSS score ≥11, which indicates severe or very severe stroke symptoms. The median NIHSS score was 38 (range 12–40) for patients who died during their stay and 11 (range 2–40) for the survivors. Seventeen patients had a depressed level of consciousness at admission, of whom swallowing function was not testable for nine.

**Table 1:** Socio-demographic and clinical characteristics of the study participants at baseline

Characteristics	n = 30
Age in years, median (IQR)	62.5 (58–67)
Sex	
Male	16 (53%)
Female	14 (47%)
Relationship status	
Married/cohabitant	21 (70%)
Widow(er)	7 (23%)
Single	2 (7%)
Living alone	
Yes	3 (10%)
No	27 (90%)
Piped water inside dwelling	
Yes	3 (10%)
No	27 (90%)
Literate	
Yes	29 (97%)
No	1 (3%)
Education	
Minimal	6 (20%)
Primary school	12 (40%)
Junior secondary school	7 (23%)
Senior secondary school	3 (10%)
Unknown	2 (7%)
Employment	
Farmer	15 (50%)
Retired	8 (27%)
Unemployed	4 (13%)
Employee	3 (10%)
Disability prior to current stroke (mRS score)	
Mild (mRS 0–2)	25 (83%)
Moderate (mRS 3)	3 (10%)
Severe (mRS 4–5)	2 (7%)
Risk factors	
Hypertension	22 (73%)
Tobacco smoking	12 (40%)
Previously	9 (30%)
Currently	3 (10%)
Previous stroke	7 (23%)
Diabetes mellitus	3 (10%)
Intermittent claudication	1 (3%)
Stroke severity (NIHSS score)	
Mild (NIHSS 0–5)	5 (17%)
Moderate (NIHSS 6–10)	3 (10%)
Severe (NIHSS 11–15)	7 (23%)
Very severe (NIHSS ≥16)	15 (50%)
Level of consciousness	
Awake	13 (43%)
Drowsy	10 (33%)
No response	7 (23%)
Swallowing function	
Normal	13 (43%)
Impaired	8 (27%)
Not testable	9 (30%)
Oxfordshire Community Stroke Project (OCSP) classification	
Total anterior circulation syndrome	11 (37%)
Partial anterior circulation syndrome	8 (27%)
Posterior circulation syndrome	1 (3%)
Lacunar syndrome	5 (17%)
Unclassifiable	5 (17%)
Siriraj Stroke Score (SSS)	
Hemorrhagic stroke (SSS >1)	16 (53%)
Ischemic stroke (SSS <−1)	9 (30%)
Equivocal (SSS −1 to 1)	5 (17%)
Blood pressure (mmHg)	
Low (<100/60)	0
Normal (100/60–140/90)	6 (20%)
High (>140/90)	24 (80%)
Plasma glucose (mmol/L)^a^	
Low (<4)	0
Normal (4.0–6.9)	14 (50%)
Intermediate (7.0–11.0)	9 (32%)
High (≥11.1)	5 (18%)

*IQR* interquartile range, *mRS* modified Rankin Scale, *NIHSS* National Institutes of Health Stroke Scale

^a^Measured for 28 patients

Three patients received a CT brain scan that showed two cases with infarction and one case with hemorrhage. The SSS predicted the pathologic stroke type correctly for two of these patients. For the third, the SSS indicated a hemorrhagic stroke, but the CT scan revealed ischemic stroke.

Among the 22 patients with known hypertension, five were treated with antihypertensive drugs at admission. All three of the diabetics were diagnosed during the stay. Plasma glucose levels were measured for 28 patients. Of the 14 patients who were classified in the intermediate or high plasma glucose category, only six received an additional test. The oral glucose tolerance test was not performed for any patient. Blood hemoglobin was measured for 10 patients with a mean value of 13.6 g/dL (range 9.9–16.7). One patient reported that she was HIV negative, whereas HIV status was unknown for the remaining 29 patients. Atrial fibrillation was not recorded because of a defective ECG device. One patient reported that she had atrial fibrillation, and her heart rate was irregular and consistent with the diagnosis.

The median length of hospital stay was 3 days (range 1–16). For those mobilized during the stay, the median time to mobilization was 23 h (range 3–96).

Table 2 describes the clinical characteristics, outcomes, and secondary prevention at discharge. The in-hospital stroke mortality was 30%. The median mRS score was 5 (IQR 4–6). Out of the nine patients with an mRS of 5, seven had a considerably reduced level of consciousness. All of the survivors were discharged home. The median BI score was 45 (IQR 0–72.5).

**Table 2:** Clinical characteristics, outcomes, and secondary prevention at discharge

Entire cohort	n = 30
Mobilization out of bed	
Yes	15 (50%)
No	15 (50%)
Complications	
Stroke progression	11 (37%)
Chest infection	5 (17%)
Bedsores	2 (7%)
Seizures	1 (3%)
Urinary tract infection	0
No	14 (47%)
In-hospital stroke mortality	9 (30%)
Disability (mRS score)	
Mild (mRS 0–2)	1 (3%)
Moderate (mRS 3)	3 (10%)
Severe (mRS 4–5)	17 (57%)
Dead (mRS 6)	9 (30%)

Survivors	n = 21
Swallowing function	
Normal	13 (62%)
Impaired	0
Not testable	8 (38%)
Blood pressure (mmHg)	
Low (<100/60)	1 (5%)
Normal (100/60–140/90)	14 (67%)
High (>140/90)	6 (29%)
Functional status (BI)	
Independency/slightly dependency (BI 95–100)	2 (10%)
Moderate dependency (BI 65–90)	3 (14%)
Severe dependency (BI 25–60)	7 (33%)
Total dependency (BI 0–20)	9 (43%)
Secondary prevention	
Acetylsalicylic acid	11 (52%)
Antihypertensive drug	9 (43%)
Warfarin	0
Non-vitamin K dependent oral anticoagulant drug	0
No	9 (43%)

*BI* Barthel Index, *mRS* modified Rankin Scale

The period prevalence of stroke was 4.5% (26/575) in the Department of Medicine during October and November 2014.

## Discussion

In this study, we found that it is feasible to collect essential clinical data on stroke patients in a resource-constrained hospital. However, the limited access to brain imaging and supporting investigations provided an inadequate basis for decision making on treatment and secondary prevention.

The WHO definition of stroke was well known and used routinely at the hospital prior to the study period. The ward facilities were quite favorable for bedside assessment. Thus, the clinical scales were easily implemented. Few data on socio-demographics and medical history were missing after the interviews of the patients and next of kin. The medical records were mostly complete with some exceptions for medical history.

The study cohort consisted of mostly serious cases. Nearly three in four patients had severe or very severe stroke symptoms as measured by the NIHSS. Similar findings were reported in the Gambian and Malawian studies [10, 11]. Furthermore, more than half of the patients in our study had a depressed level of consciousness, and one-third were classified with total anterior circulation syndrome. We suggest that severe stroke events led to hospitalization more frequently than mild and moderate events. The widespread poverty might be a major cause why patients with mild and moderate stroke symptoms did not present to the hospital.

Among the risk factors available for recording, hypertension was the most frequent. This is entirely consistent with other studies in low-income SSA [9–13]. The INTERSTROKE study suggested that hypertension is the dominating risk factor for stroke, particularly in low-income regions [30]. A study conducted in Moramanga district, Madagascar, showed that hypertension is highly prevalent and insufficiently treated [31]. Besides hypertension, tobacco smoking was a common risk factor in our study. Both hypertension and tobacco smoking were easily detected. Therefore, antihypertensive treatment and strategies for smoking cessation could be implemented. The shortage of laboratory tests and absence of ECGs led to an incomplete mapping of risk factors, such as diabetes, HIV, and atrial fibrillation, and few measures were taken to modify these.

The limited access to brain imaging meant that the pathological stroke type remained unknown for the great majority of patients. By using the SSS, approximately half of the patients were classified as having a hemorrhagic stroke. However, the SSS has not proven to be reliable enough to distinguish between ischemic and hemorrhagic stroke [32]. Nevertheless, clinical assessment could be used to determine which pathological stroke type that is most likely [33]. The presence of headache, vomiting, severe hypertension, neck stiffness, and coma are suggestive of hemorrhagic stroke. When these symptoms and findings are absent, it should be beneficial to initiate acetylsalicylic acid for secondary prevention after stroke with unknown etiology [34]. However, antithrombotic treatments such as thrombolysis and anticoagulation cannot be administrated without brain imaging.

Chest infection was the most frequent complication of immobility in our study. Complications of immobility are potentially preventable [27]. A few basic measures to prevent infections and bedsores should be feasible in resource-constrained settings: testing of swallowing function before giving the patient oral feedings, avoiding indwelling urinary catheter whenever possible, repositioning the patient in bed every 2 h, and using cushions to protect bony areas. Early mobilization is a key component of acute stroke care [35]. We experienced considerable uncertainty about how to mobilize the patient, both among the staff and family members. Therefore, we suggest that guidelines for mobilization should be handed out to all caregivers.

The in-hospital stroke mortality of 30% was within the expected range in light of the results from the Gambian, Malawian, and Mozambican studies [10–12].

At discharge, most of the survivors had a severe disability (mRS 4–5) and severe or total dependency (BI ≤ 60). Similar findings for disability have been reported in Ethiopia and for dependency in the Gambia [9, 10].

A few previous studies have also reported on stroke treatment in hospitals of SSA without full access to technical facilities. The Gambian study was conducted in the absence of CT scans [10]. Some limitations in access to ECGs and CT scans were described in Malawi [11]. However, the shortage of laboratory tests and absence of ECGs seemed unique to our study.

The limitations of our study are the small sample size, the wide variation in prehospital time lapse, and no long-term follow-up information. We cannot generalize on the basis of this small-scale study conducted at a single hospital. However, the general conditions at the Andranomadio Lutheran Hospital are quite similar to those at many other hospitals in Madagascar.

In our study, there was probably a bias towards serious cases. Nevertheless, we are concerned about the high in-hospital mortality and the low functional status for the survivors at discharge. We suppose that both the lack of a systematic approach to stroke care and the limited access to technical facilities contributed to the severe outcomes. The clinical examinations at admission provided a basis for supportive treatment, such as intravenous fluids, paracetamol for pyrexia, and reduction of very elevated blood pressure [14]. However, there was no structured protocol for the management of physiological abnormalities in the acute phase. Moreover, several risk factors and complications were detected, but few measures were taken to modify or treat them. Finally, socioeconomic status had a major impact on the possibility of conducting a CT brain scan. This examination was unaffordable to most of the patients, and this circumstance appears unlikely to change in the next few years. These conditions illustrate the enormous gap between high-income and low-income countries and emphasize the urgent need for guidelines for the management of acute stroke of unknown etiology in resource-limited settings [36].

Based on our experiences, there is a long way to go to establish modern stroke services in resource-constrained hospitals in Madagascar. Nevertheless, we think it is possible to take small steps towards a better in-hospital stroke care. When access to brain imaging and supporting investigations are limited, the care for stroke patients will largely rely on non-drug treatments and rehabilitation interventions [16]. Involvement of the carers, particularly family caregivers, will be a central component of the service. We suggest introducing a practical protocol for stroke assessment and management. This will ensure a systematic approach to patients hospitalized for stroke. The protocol should include stabilization at admission, diagnostic assessment, assessment of stroke severity, management of risk factors, prevention and management of complications, mobilization, assessment of disability, mapping of home circumstances, strategies for secondary prevention, and education programs for family caregivers. The measures must be adapted to the resources available to the patient. It is also crucial that a stroke assessment and management protocol does not lead to increasing out-of-pocket costs for the patient.

Our study suggests that it is possible to collect data required for establishing a basic stroke assessment and management protocol in a resource-constrained hospital in Madagascar. Such a protocol could be the first step towards improving the in-hospital stroke care [16]. The study also suggests that further research on stroke services in resource-constrained settings is feasible.

## Conclusions

This small-scale study was performed in a resource-constrained hospital in Madagascar. We collected essential clinical parameters from stroke patients, despite the lack of technical facilities. We found that most patients had severe symptoms at admission and serious early outcomes. The study suggests that it is feasible to register crucial stroke data in a resource-constrained setting, which should be useful for planning of future stroke services and research projects.

## Abbreviations

BI: Barthel Index
CT: computed tomography
ECG: electrocardiography
HIV: human immunodeficiency virus
IQR: interquartile range
mRS: modified Rankin Scale
NIHSS: National Institutes of Health Stroke Scale
OCSP: Oxfordshire Community Stroke Project
SSA: sub-Saharan Africa
SSS: Siriraj Stroke Score
WHO: World Health Organization
